# 1-Cyclo­hexyl­methyl-3-methyl-2-[(phenyl­imino)(sulfido)meth­yl]benzimidazolium

**DOI:** 10.1107/S1600536808008842

**Published:** 2008-04-10

**Authors:** Mehmet Akkurt, Selvi Karaca, Hasan Küçükbay, Nihat Şireci, Orhan Büyükgüngör

**Affiliations:** a Department of Physics, Faculty of Arts and Sciences, Erciyes University, 38039 Kayseri, Turkey; b Department of Chemistry, Faculty of Arts and Sciences, Ínönü University, 44280 Malatya, Turkey; c Department of Chemistry, Faculty of Arts and Sciences, Adıyaman University, 02040 Adıyaman, Turkey; d Department of Physics, Faculty of Arts and Sciences, Ondokuz Mayıs University, 55139 Samsun, Turkey

## Abstract

In the zwitterionic title compound, C_22_H_25_N_3_S, the benzimid­azole ring system makes a dihedral angle of 55.69 (11)° with the phenyl ring. In the crystal structure, inter- and intra­molecular C—H⋯S inter­actions occur.

## Related literature

For related structures, see: Öztürk *et al.* (2004 ▶); Akkurt *et al.* (2005 ▶). For background, see: Allen *et al.* (1987 ▶); Cremer & Pople (1975 ▶); Küçükbay *et al.* (1995 ▶); Winberg & Coffman (1965 ▶).
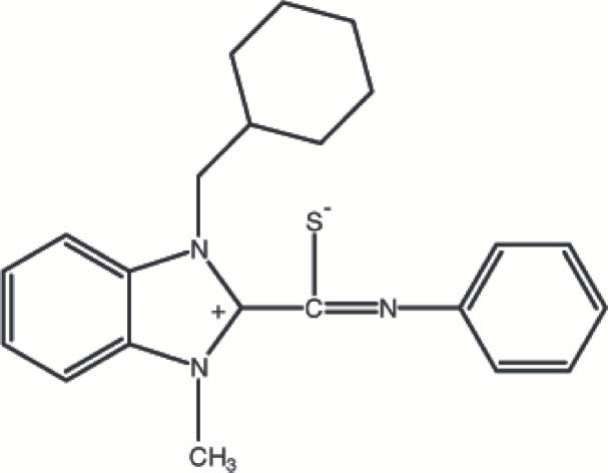


## Experimental

### 

#### Crystal data

C_22_H_25_N_3_S
*M* *_r_* = 363.52
Monoclinic, 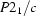

*a* = 11.0999 (8) Å
*b* = 12.4601 (7) Å
*c* = 15.0143 (14) Å
β = 99.007 (7)°
*V* = 2051.0 (3) Å^3^
*Z* = 4
Mo *K*α radiation
μ = 0.17 mm^−1^
*T* = 296 K
0.62 × 0.56 × 0.51 mm

#### Data collection

Stoe IPDSII diffractometer
Absorption correction: integration (*X-RED32*; Stoe & Cie, 2002 ▶) *T*_min_ = 0.903, *T*_max_ = 0.919
11769 measured reflections
3997 independent reflections
2727 reflections with *I* > 2σ(*I*)
*R*_int_ = 0.031

#### Refinement

*R*[*F* ^2^ > 2σ(*F* ^2^)] = 0.046
*wR*(*F* ^2^) = 0.127
*S* = 1.02
3997 reflections
236 parameters
H-atom parameters constrained
Δρ_max_ = 0.36 e Å^−3^
Δρ_min_ = −0.37 e Å^−3^

### 

Data collection: *X-AREA* (Stoe & Cie, 2002 ▶); cell refinement: *X-AREA*; data reduction: *X-RED32* (Stoe & Cie, 2002 ▶); program(s) used to solve structure: *SIR97* (Altomare *et al.*, 1999 ▶); program(s) used to refine structure: *SHELXL97* (Sheldrick, 2008 ▶); molecular graphics: *ORTEP-3 for Windows* (Farrugia, 1997 ▶); software used to prepare material for publication: *WinGX* (Farrugia, 1999 ▶).

## Supplementary Material

Crystal structure: contains datablocks global, I. DOI: 10.1107/S1600536808008842/hb2714sup1.cif

Structure factors: contains datablocks I. DOI: 10.1107/S1600536808008842/hb2714Isup2.hkl

Additional supplementary materials:  crystallographic information; 3D view; checkCIF report

## Figures and Tables

**Table 1:** Hydrogen-bond geometry (Å, °)

*D*—H⋯*A*	*D*—H	H⋯*A*	*D*⋯*A*	*D*—H⋯*A*
C16—H16*A*⋯S1^i^	0.97	2.73	3.683 (2)	167
C16—H16*B*⋯S1	0.97	2.78	3.451 (2)	127

Symmetry code: (i) 
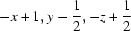
.

**Fig. 1.**
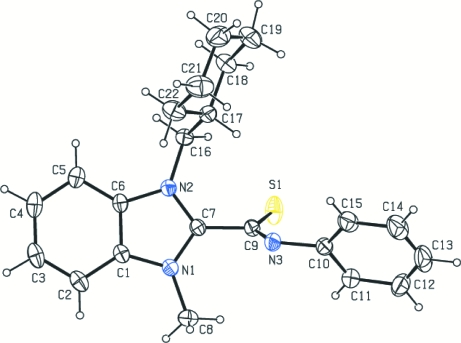
View of the molecular structure of (I) showing 20% displacement ellipsoids for the non-H atoms.

**Fig. 2.**
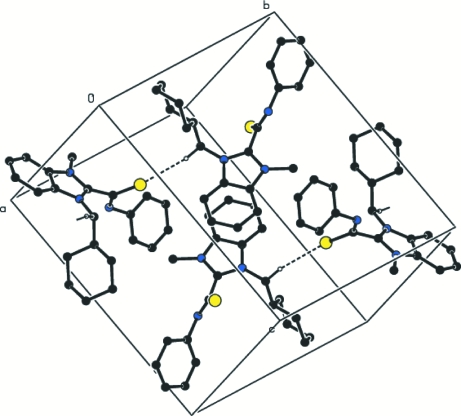
View of the packing and intermolecular C—H···S hydrogen bond contacts in the unit cell of (I).

## supplementary crystallographic information

### Comment

Electron-rich olefins are exteremly reactive, powerful π-bases, which are readily converted by aryl isothiocyanates to stable yellow-colored mercapto-*N*-arylformimidoylimidazolinium orbenzimidazolinium inner salts (zwitterions) in high yield (Winberg & Coffman, 1965; Küçükbay *et al.*, 1995).  As part of our onging studies of such materials (Öztürk *et al.*, 2004; Akkurt *et al.*, 2005), we now report the synthesis and structure of the title compound, (I).

The S1—C9 formal single bond of length of 1.6968 (18) Å in (I) is comparable to those reported for similar structures (Öztürk *et al.*, 2004; Akkurt *et al.*, 2005). Otherwise the bond lengths and angles in (I) are normal (Allen *et al.*,  1987).  The benzimidazole ring system (N1/N2/C1–C7) is almost planar, with maximum deviations of -0.012 (2) Å for C1 and C6, and makes a dihedral angle of 55.69 (11)° with the phenyl ring (C10–C15). The cyclohexane ring system (C17–C22) has a normal chair conformation [puckering parameters (Cremer & Pople, 1975) are Q_T_ = 0.554 (3) Å, θ = 175.9 (3)°, φ = 136 (4)°].

In the crystal of (I), the molecules display inter- and intramolecular C—H···S interactions (Table 1, Fig. 2).

### Experimental

Phenyl isothiocyanate (0.7 ml, 5. 86 mmol) was added to a solution of bis(1-cyclohexylmethyl-3-methylbenzimidazolidine-2-ylidene) (1.1 g, 2.41 mmol) in toluene (15 ml) and the mixture was stirred at room temperature for 2 h. A yellow solid was precipitated in solution. The precipitate was filtered and recrystallized in EtOH / DMF to yield yellow blocks of (I). (Yield: 1.49 g, 85%. m.p.:463–465 K). ^1^H NMR (DMSO-d_6_,δ, p.p.m.): 1.11 (s, ring methylene, 6H), 1.59 (d, ring methylene, 4H), 2.09 (s, ring methylene, 1H), 4.04 (s, CH_3_, 3H), 4.45 (d, –CH_2_—N, 2H), 7.22 (m, Ar—H (forPhNCS), 5H), 7.93 (m, Ar—H, 4H); ^13^C NMR (DMSO-d_6_, δ,p.p.m.): 25.10, 25.15, 30.05, 30.12,38.05, 50.03, 112.11, 122.04, 122.10, 126.01, 129.03, 129.14, 131.23, 149.06,151.34, 167.44. Analysis calculated for C_22_H_25_N_3_S: C 72.72, H 6.88, N 11.57, S 8.81%; found: C 71.82, H 6.87, N 11.46, S 8.22%.

### Refinement

All H atoms were placed in calculated positions and refined as riding with C—H = 0.93–0.97 Å and *U*_iso_(H) = 1.2 or 1.5*U*_eq_(C).

### Crystal data

**Table:**

C_22_H_25_N_3_S	*F*_000_ = 776
*M**_r_* = 363.52	*D*_x_ = 1.177 Mg m^−^^3^
Monoclinic, *P*2_1_/*c*	Mo *K*α radiation λ = 0.71073 Å
Hall symbol: -P 2ybc	Cell parameters from 12543 reflections
*a* = 11.0999 (8) Å	θ = 1.4–28.0º
*b* = 12.4601 (7) Å	µ = 0.17 mm^−^^1^
*c* = 15.0143 (14) Å	*T* = 296 K
β = 99.007 (7)º	Block, yellow
*V* = 2051.0 (3) Å^3^	0.62 × 0.56 × 0.51 mm
*Z* = 4	

### Data collection

**Table:**

Stoe IPDSII diffractometer	3997 independent reflections
Monochromator: plane graphite	2727 reflections with *I* > 2σ(*I*)
Detector resolution: 6.67 pixels mm^-1^	*R*_int_ = 0.031
*T* = 296 K	θ_max_ = 26.0º
ω scans	θ_min_ = 1.9º
Absorption correction: integration(X-RED32; Stoe & Cie, 2002)	*h* = −13→13
*T*_min_ = 0.903, *T*_max_ = 0.919	*k* = −15→13
11769 measured reflections	*l* = −18→18

### Refinement

**Table:**

Refinement on *F*^2^	Secondary atom site location: difference Fourier map
Least-squares matrix: full	Hydrogen site location: inferred from neighbouring sites
*R*[*F*^2^ > 2σ(*F*^2^)] = 0.046	H-atom parameters constrained
*wR*(*F*^2^) = 0.127	*w* = 1/[σ^2^(*F*_o_^2^) + (0.0746*P*)^2^] where *P* = (*F*_o_^2^ + 2*F*_c_^2^)/3
*S* = 1.02	(Δ/σ)_max_ < 0.001
3997 reflections	Δρ_max_ = 0.36 e Å^−^^3^
236 parameters	Δρ_min_ = −0.37 e Å^−^^3^
Primary atom site location: structure-invariant direct methods	Extinction correction: none

### Special details

**Table:**

Geometry. Bond distances, angles *etc*. have been calculated using the rounded fractional coordinates. All su's are estimated from the variances of the (full) variance-covariance matrix. The cell e.s.d.'s are taken into account in the estimation of distances, angles and torsion angles
Refinement. Refinement on *F*^2^ for ALL reflections except those flagged by the user for potential systematic errors. Weighted *R*-factors *wR* and all goodnesses of fit *S* are based on *F*^2^, conventional *R*-factors *R* are based on *F*, with *F* set to zero for negative *F*^2^. The observed criterion of *F*^2^ > σ(*F*^2^) is used only for calculating -*R*-factor-obs *etc*. and is not relevant to the choice of reflections for refinement. *R*-factors based on *F*^2^ are statistically about twice as large as those based on *F*, and *R*-factors based on ALL data will be even larger.

### Fractional atomic coordinates and isotropic or equivalent isotropic displacement parameters (Å^2^)

**Table:**

	*x*	*y*	*z*	*U*_iso_*/*U*_eq_	
S1	0.40319 (5)	0.79425 (5)	0.26689 (5)	0.0886 (2)	
N1	0.28968 (12)	0.63297 (12)	0.44357 (9)	0.0502 (5)	
N2	0.34915 (12)	0.54355 (11)	0.33351 (9)	0.0487 (4)	
N3	0.16469 (13)	0.75498 (13)	0.28359 (10)	0.0572 (5)	
C1	0.32300 (14)	0.53150 (14)	0.47690 (11)	0.0503 (6)	
C2	0.32500 (17)	0.48719 (19)	0.56177 (13)	0.0650 (7)	
C3	0.36543 (19)	0.3831 (2)	0.57255 (16)	0.0738 (8)	
C4	0.40346 (18)	0.32577 (18)	0.50294 (17)	0.0738 (8)	
C5	0.40103 (17)	0.36931 (16)	0.41825 (15)	0.0632 (7)	
C6	0.36035 (14)	0.47437 (14)	0.40713 (12)	0.0494 (5)	
C7	0.30640 (14)	0.63806 (14)	0.35726 (11)	0.0465 (5)	
C8	0.2483 (2)	0.72060 (18)	0.49579 (14)	0.0687 (7)	
C9	0.27958 (15)	0.73442 (14)	0.29906 (11)	0.0507 (5)	
C10	0.11976 (17)	0.84557 (17)	0.23179 (13)	0.0614 (6)	
C11	0.1515 (2)	0.94910 (19)	0.25693 (16)	0.0791 (8)	
C12	0.0936 (3)	1.0351 (2)	0.2082 (2)	0.1043 (11)	
C13	0.0082 (3)	1.0155 (4)	0.1341 (3)	0.1179 (14)	
C14	−0.0228 (3)	0.9139 (4)	0.1091 (2)	0.1084 (13)	
C15	0.03093 (19)	0.8291 (2)	0.15829 (15)	0.0815 (9)	
C16	0.38428 (16)	0.51876 (17)	0.24565 (12)	0.0577 (6)	
C17	0.27793 (17)	0.49794 (17)	0.17240 (12)	0.0612 (7)	
C18	0.3246 (2)	0.4812 (2)	0.08393 (14)	0.0816 (9)	
C19	0.2221 (3)	0.4596 (3)	0.00661 (17)	0.1112 (15)	
C20	0.1411 (3)	0.3698 (3)	0.02668 (19)	0.1048 (11)	
C21	0.0958 (3)	0.3852 (3)	0.1148 (2)	0.1050 (11)	
C22	0.2009 (2)	0.4028 (2)	0.19119 (16)	0.0819 (9)	
H2	0.30040	0.52580	0.60880	0.0780*	
H3	0.36730	0.35010	0.62830	0.0890*	
H4	0.43140	0.25590	0.51360	0.0890*	
H5	0.42530	0.33040	0.37130	0.0760*	
H8A	0.25970	0.78770	0.46680	0.0830*	
H8B	0.29450	0.72060	0.55540	0.0830*	
H8C	0.16330	0.71110	0.49940	0.0830*	
H11	0.21140	0.96190	0.30640	0.0950*	
H12	0.11300	1.10520	0.22600	0.1250*	
H13	−0.02890	1.07270	0.10060	0.1410*	
H14	−0.08080	0.90140	0.05830	0.1300*	
H15	0.00700	0.75950	0.14170	0.0980*	
H16A	0.43660	0.45600	0.25200	0.0690*	
H16B	0.43130	0.57830	0.22750	0.0690*	
H17	0.22580	0.56180	0.16650	0.0730*	
H18A	0.38080	0.42110	0.08990	0.0980*	
H18B	0.36900	0.54460	0.07030	0.0980*	
H19A	0.17360	0.52420	−0.00590	0.1330*	
H19B	0.25680	0.44200	−0.04700	0.1330*	
H20A	0.18580	0.30280	0.02830	0.1260*	
H20B	0.07190	0.36490	−0.02140	0.1260*	
H21A	0.04980	0.32240	0.12770	0.1260*	
H21B	0.04160	0.44660	0.11050	0.1260*	
H22A	0.16900	0.41490	0.24690	0.0980*	
H22B	0.25140	0.33890	0.19880	0.0980*	

### Atomic displacement parameters (Å^2^)

**Table:**

	*U*^11^	*U*^22^	*U*^33^	*U*^12^	*U*^13^	*U*^23^
S1	0.0631 (3)	0.0819 (4)	0.1212 (5)	−0.0041 (3)	0.0155 (3)	0.0461 (4)
N1	0.0531 (7)	0.0518 (9)	0.0451 (8)	−0.0037 (6)	0.0063 (6)	0.0022 (7)
N2	0.0498 (7)	0.0496 (8)	0.0461 (8)	0.0028 (6)	0.0061 (6)	0.0030 (6)
N3	0.0568 (8)	0.0579 (9)	0.0562 (9)	0.0096 (7)	0.0069 (7)	0.0065 (7)
C1	0.0478 (8)	0.0543 (11)	0.0468 (10)	−0.0099 (7)	0.0015 (7)	0.0064 (8)
C2	0.0643 (11)	0.0787 (14)	0.0501 (11)	−0.0184 (10)	0.0035 (8)	0.0113 (10)
C3	0.0692 (12)	0.0802 (16)	0.0676 (13)	−0.0205 (11)	−0.0033 (10)	0.0307 (12)
C4	0.0626 (11)	0.0596 (12)	0.0941 (17)	−0.0073 (9)	−0.0038 (11)	0.0271 (12)
C5	0.0598 (10)	0.0516 (11)	0.0762 (13)	−0.0010 (8)	0.0049 (9)	0.0094 (10)
C6	0.0446 (8)	0.0500 (10)	0.0515 (10)	−0.0047 (7)	0.0008 (7)	0.0094 (8)
C7	0.0443 (8)	0.0503 (10)	0.0441 (9)	−0.0025 (7)	0.0047 (6)	0.0023 (7)
C8	0.0832 (13)	0.0683 (14)	0.0568 (11)	0.0026 (10)	0.0175 (9)	−0.0095 (10)
C9	0.0565 (9)	0.0501 (10)	0.0443 (9)	0.0013 (7)	0.0044 (7)	0.0030 (8)
C10	0.0633 (10)	0.0690 (13)	0.0535 (10)	0.0197 (9)	0.0143 (8)	0.0077 (10)
C11	0.1043 (16)	0.0682 (15)	0.0667 (13)	0.0194 (12)	0.0196 (12)	0.0080 (11)
C12	0.133 (2)	0.0744 (17)	0.117 (2)	0.0412 (16)	0.055 (2)	0.0263 (16)
C13	0.110 (2)	0.147 (3)	0.104 (2)	0.073 (2)	0.0394 (18)	0.062 (2)
C14	0.0856 (17)	0.159 (3)	0.0791 (17)	0.052 (2)	0.0083 (13)	0.029 (2)
C15	0.0686 (12)	0.1101 (19)	0.0644 (13)	0.0266 (12)	0.0061 (10)	0.0061 (13)
C16	0.0604 (10)	0.0609 (12)	0.0541 (10)	0.0051 (8)	0.0165 (8)	0.0017 (9)
C17	0.0694 (11)	0.0609 (12)	0.0528 (11)	0.0152 (9)	0.0077 (8)	−0.0019 (9)
C18	0.1018 (16)	0.0905 (17)	0.0537 (12)	0.0079 (13)	0.0156 (11)	0.0038 (12)
C19	0.141 (3)	0.133 (3)	0.0545 (14)	0.023 (2)	−0.0003 (15)	0.0003 (15)
C20	0.114 (2)	0.105 (2)	0.0840 (18)	0.0193 (17)	−0.0204 (16)	−0.0297 (16)
C21	0.0878 (16)	0.127 (2)	0.0957 (19)	−0.0097 (15)	0.0007 (14)	−0.0336 (18)
C22	0.0780 (14)	0.0992 (18)	0.0685 (14)	−0.0149 (12)	0.0116 (11)	−0.0101 (13)

### Geometric parameters (Å, °)

**Table:**

S1—C9	1.6968 (18)	C20—C21	1.500 (4)
N1—C1	1.388 (2)	C21—C22	1.518 (4)
N1—C7	1.339 (2)	C2—H2	0.9300
N1—C8	1.460 (3)	C3—H3	0.9300
N2—C6	1.392 (2)	C4—H4	0.9300
N2—C7	1.339 (2)	C5—H5	0.9300
N2—C16	1.466 (2)	C8—H8A	0.9600
N3—C9	1.286 (2)	C8—H8B	0.9600
N3—C10	1.416 (3)	C8—H8C	0.9600
C1—C2	1.386 (3)	C11—H11	0.9300
C1—C6	1.383 (2)	C12—H12	0.9300
C2—C3	1.374 (3)	C13—H13	0.9300
C3—C4	1.385 (3)	C14—H14	0.9300
C4—C5	1.379 (3)	C15—H15	0.9300
C5—C6	1.386 (3)	C16—H16A	0.9700
C7—C9	1.487 (2)	C16—H16B	0.9700
C10—C11	1.374 (3)	C17—H17	0.9800
C10—C15	1.375 (3)	C18—H18A	0.9700
C11—C12	1.396 (4)	C18—H18B	0.9700
C12—C13	1.366 (5)	C19—H19A	0.9700
C13—C14	1.350 (7)	C19—H19B	0.9700
C14—C15	1.371 (5)	C20—H20A	0.9700
C16—C17	1.505 (3)	C20—H20B	0.9700
C17—C18	1.514 (3)	C21—H21A	0.9700
C17—C22	1.514 (3)	C21—H21B	0.9700
C18—C19	1.517 (4)	C22—H22A	0.9700
C19—C20	1.495 (5)	C22—H22B	0.9700
			
C1—N1—C7	108.81 (14)	N1—C8—H8B	110.00
C1—N1—C8	125.17 (14)	N1—C8—H8C	109.00
C7—N1—C8	125.97 (15)	H8A—C8—H8B	110.00
C6—N2—C7	108.94 (14)	H8A—C8—H8C	109.00
C6—N2—C16	125.52 (15)	H8B—C8—H8C	109.00
C7—N2—C16	125.48 (15)	C10—C11—H11	120.00
C9—N3—C10	120.80 (16)	C12—C11—H11	120.00
N1—C1—C2	131.24 (17)	C11—C12—H12	120.00
N1—C1—C6	106.88 (14)	C13—C12—H12	120.00
C2—C1—C6	121.87 (17)	C12—C13—H13	120.00
C1—C2—C3	116.26 (19)	C14—C13—H13	120.00
C2—C3—C4	122.0 (2)	C13—C14—H14	120.00
C3—C4—C5	121.9 (2)	C15—C14—H14	120.00
C4—C5—C6	116.21 (19)	C10—C15—H15	120.00
N2—C6—C1	106.42 (15)	C14—C15—H15	120.00
N2—C6—C5	131.86 (17)	N2—C16—H16A	109.00
C1—C6—C5	121.70 (17)	N2—C16—H16B	109.00
N1—C7—N2	108.95 (15)	C17—C16—H16A	109.00
N1—C7—C9	124.16 (15)	C17—C16—H16B	109.00
N2—C7—C9	126.89 (15)	H16A—C16—H16B	108.00
S1—C9—N3	133.19 (14)	C16—C17—H17	108.00
S1—C9—C7	115.18 (12)	C18—C17—H17	108.00
N3—C9—C7	111.63 (15)	C22—C17—H17	108.00
N3—C10—C11	122.96 (18)	C17—C18—H18A	109.00
N3—C10—C15	117.92 (19)	C17—C18—H18B	109.00
C11—C10—C15	118.8 (2)	C19—C18—H18A	109.00
C10—C11—C12	120.0 (2)	C19—C18—H18B	109.00
C11—C12—C13	119.6 (3)	H18A—C18—H18B	108.00
C12—C13—C14	120.6 (4)	C18—C19—H19A	109.00
C13—C14—C15	120.2 (3)	C18—C19—H19B	109.00
C10—C15—C14	120.9 (3)	C20—C19—H19A	109.00
N2—C16—C17	113.91 (15)	C20—C19—H19B	109.00
C16—C17—C18	109.15 (16)	H19A—C19—H19B	108.00
C16—C17—C22	113.59 (17)	C19—C20—H20A	109.00
C18—C17—C22	109.66 (18)	C19—C20—H20B	109.00
C17—C18—C19	112.2 (2)	C21—C20—H20A	109.00
C18—C19—C20	112.4 (2)	C21—C20—H20B	109.00
C19—C20—C21	112.2 (3)	H20A—C20—H20B	108.00
C20—C21—C22	111.2 (3)	C20—C21—H21A	109.00
C17—C22—C21	111.2 (2)	C20—C21—H21B	109.00
C1—C2—H2	122.00	C22—C21—H21A	109.00
C3—C2—H2	122.00	C22—C21—H21B	109.00
C2—C3—H3	119.00	H21A—C21—H21B	108.00
C4—C3—H3	119.00	C17—C22—H22A	109.00
C3—C4—H4	119.00	C17—C22—H22B	109.00
C5—C4—H4	119.00	C21—C22—H22A	109.00
C4—C5—H5	122.00	C21—C22—H22B	109.00
C6—C5—H5	122.00	H22A—C22—H22B	108.00
N1—C8—H8A	109.00		
			
C7—N1—C1—C2	178.50 (18)	C1—C2—C3—C4	0.7 (3)
C8—N1—C1—C2	0.9 (3)	C2—C3—C4—C5	−1.1 (3)
C7—N1—C1—C6	−0.28 (18)	C3—C4—C5—C6	1.1 (3)
C8—N1—C1—C6	−177.88 (16)	C4—C5—C6—C1	−0.8 (3)
C1—N1—C7—N2	0.09 (19)	C4—C5—C6—N2	177.97 (18)
C8—N1—C7—N2	177.66 (16)	N2—C7—C9—N3	112.86 (19)
C1—N1—C7—C9	179.56 (15)	N1—C7—C9—S1	113.46 (16)
C8—N1—C7—C9	−2.9 (3)	N1—C7—C9—N3	−66.5 (2)
C6—N2—C7—N1	0.14 (18)	N2—C7—C9—S1	−67.2 (2)
C16—N2—C7—N1	−177.02 (15)	N3—C10—C11—C12	−172.9 (2)
C6—N2—C7—C9	−179.31 (15)	C11—C10—C15—C14	1.9 (3)
C16—N2—C7—C9	3.5 (3)	C15—C10—C11—C12	0.2 (3)
C6—N2—C16—C17	105.6 (2)	N3—C10—C15—C14	175.3 (2)
C7—N2—C16—C17	−77.7 (2)	C10—C11—C12—C13	−2.0 (4)
C7—N2—C6—C5	−179.20 (18)	C11—C12—C13—C14	1.7 (5)
C7—N2—C6—C1	−0.31 (18)	C12—C13—C14—C15	0.3 (5)
C16—N2—C6—C1	176.85 (15)	C13—C14—C15—C10	−2.2 (4)
C16—N2—C6—C5	−2.0 (3)	N2—C16—C17—C18	176.01 (17)
C9—N3—C10—C11	−61.4 (3)	N2—C16—C17—C22	−61.3 (2)
C9—N3—C10—C15	125.6 (2)	C16—C17—C18—C19	180.0 (2)
C10—N3—C9—S1	−2.0 (3)	C22—C17—C18—C19	54.9 (3)
C10—N3—C9—C7	178.02 (15)	C16—C17—C22—C21	−179.6 (2)
N1—C1—C6—N2	0.36 (18)	C18—C17—C22—C21	−57.1 (3)
C6—C1—C2—C3	−0.4 (3)	C17—C18—C19—C20	−52.9 (3)
N1—C1—C2—C3	−179.03 (18)	C18—C19—C20—C21	52.0 (4)
N1—C1—C6—C5	179.38 (16)	C19—C20—C21—C22	−54.1 (4)
C2—C1—C6—N2	−178.56 (16)	C20—C21—C22—C17	57.1 (3)
C2—C1—C6—C5	0.5 (3)		

### Hydrogen-bond geometry (Å, °)

**Table:**

*D*—H···*A*	*D*—H	H···*A*	*D*···*A*	*D*—H···*A*
C16—H16A···S1^i^	0.97	2.73	3.683 (2)	167
C16—H16B···S1	0.97	2.78	3.451 (2)	127

Symmetry codes: (i) −*x*+1, *y*−1/2, −*z*+1/2.
